# Stomatal Opening: The Role of Cell-Wall Mechanical Anisotropy and Its Analytical Relations to the Bio-composite Characteristics

**DOI:** 10.3389/fpls.2017.02061

**Published:** 2017-12-12

**Authors:** Ziv Marom, Ilana Shtein, Benny Bar-On

**Affiliations:** Department of Mechanical Engineering, Ben-Gurion University of the Negev, Beer-Sheva, Israel

**Keywords:** stomatal mechanics, bio-composites, finite-element modeling, multi-scale modeling, bio-mechanics, plant mechanics, bio-mechanical actuators, stomatal opening

## Abstract

Stomata are pores on the leaf surface, which are formed by a pair of curved, tubular guard cells; an increase in turgor pressure deforms the guard cells, resulting in the opening of the stomata. Recent studies employed numerical simulations, based on experimental data, to analyze the effects of various structural, chemical, and mechanical features of the guard cells on the stomatal opening characteristics; these studies all support the well-known qualitative observation that the mechanical anisotropy of the guard cells plays a critical role in stomatal opening. Here, we propose a computationally based analytical model that quantitatively establishes the relations between the degree of anisotropy of the guard cell, the bio-composite constituents of the cell wall, and the aperture and area of stomatal opening. The model introduces two non-dimensional key parameters that dominate the guard cell deformations—the inflation driving force and the anisotropy ratio—and it serves as a generic framework that is not limited to specific plant species. The modeling predictions are in line with a wide range of previous experimental studies, and its analytical formulation sheds new light on the relations between the structure, mechanics, and function of stomata. Moreover, the model provides an analytical tool to back-calculate the elastic characteristics of the matrix that composes the guard cell walls, which, to the best of our knowledge, cannot be probed by direct nano-mechanical experiments; indeed, the estimations of our model are in good agreement with recently published results of independent numerical optimization schemes. The emerging insights from the stomatal structure-mechanics “design guidelines” may promote the development of miniature, yet complex, multiscale composite actuation mechanisms for future engineering platforms.

## Introduction

**Figure 1:**
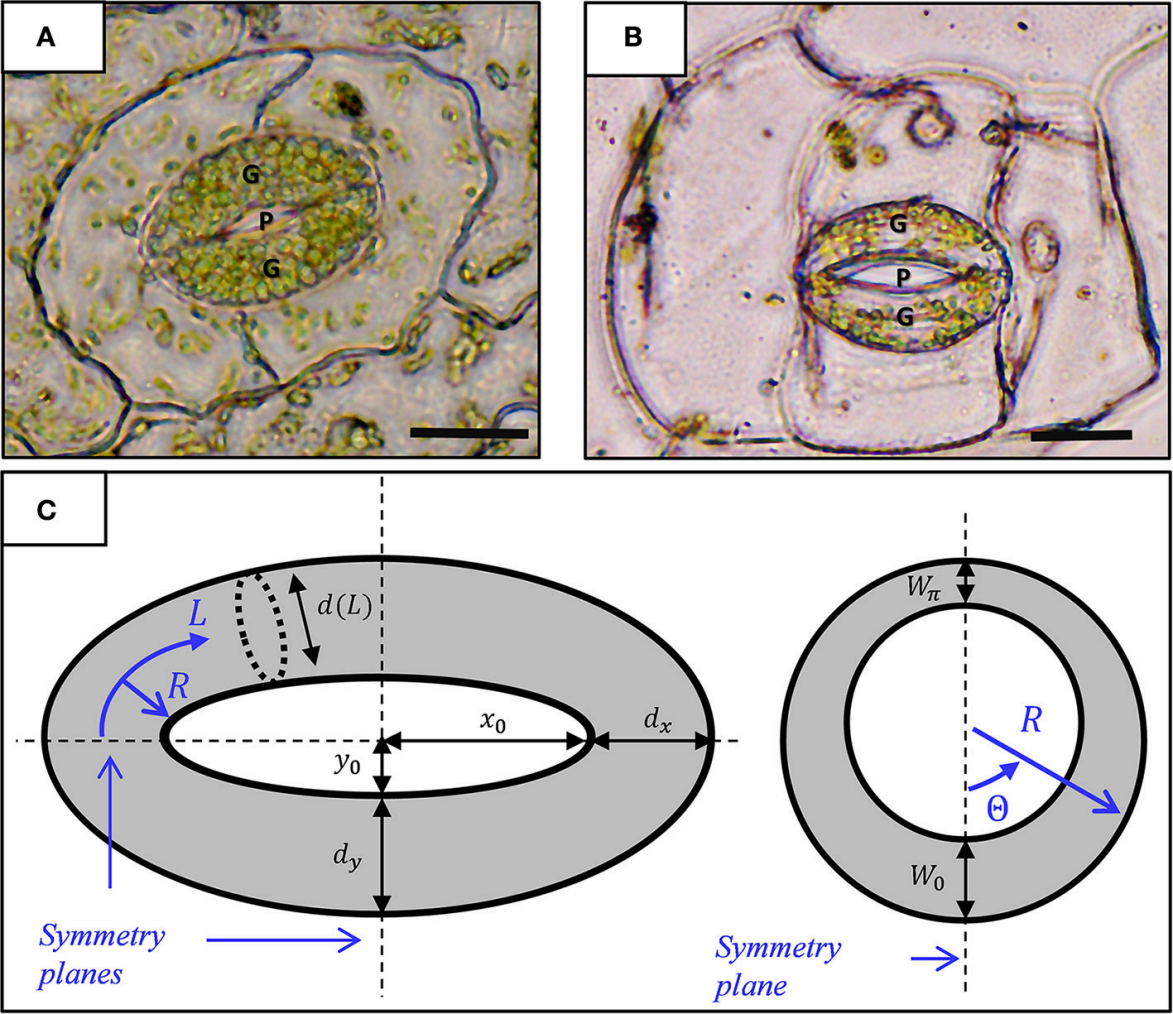
Micrograph showing a stoma of the fern *Nephrolepis exaltata* **(A)** and the angiosperm *Tradescantiapallida* **(B)**. A typical kidney-shaped stoma comprises two guard cells (G) with a pore (P) between them. Scale-−10 μm. **(C)** Geometric model for a stoma: top view (left) and radial section (right). The stomatal pore and guard cells are enclosed by elliptical contours of radii (*x*_0_, *y*_0_) and (*x*_0_+*d*_*x*_, *y*_0_+*d*_*y*_), respectively. *L* and *R* represent the stomatal longitudinal and radial directions (curvilinear coordinates), respectively. The interior of the guard cell with inner diameter *d*(*L*) is enclosed by a non-uniform wall of thickness *W*_0_+(*W*_π_−*W*_0_)·Θ/π; note that *d*(*L*)≫*W*(Θ).

Stomata are pores on the surfaces of leaves that function as bio-mechanical valves which control gas exchange in plants (Raven et al., 2005). Each stoma is bordered by a pair of curved, tubular guard cells (see Figure 1) that work together to control stomatal opening and closing. Their flexible and robust structure enable stomata to reversibly open multiple times daily, a process that is driven by increasing the water pressure (turgor) in the guard cells, thus causing them to undergo significant, yet reversible, shape changes that effectively increase the stomatal pore area between the guard cells (Zeiger et al., 1987). These shape changes include extensive elongation that, associated with limited cross-sectional expansions, is possible due to the high circumferential stiffness of the guard cell wall relative to its lower stiffness in the other directions (Aylor et al., 1973). From a material-level perspective, the high mechanical anisotropy of plant tissues is conferred by their cell walls bio-composite microstructure, which combines stiff cellulose microfibrils with a softer matrix material (pectin-rich hemicellulose, lignin, and some structural protein etc.; Jones et al., 2003; Fratzl and Weinkamer, 2007; Meyers et al., 2008; Doblin et al., 2010; Gibson et al., 2010; Bar-On et al., 2014). The anisotropic nature of stomatal cell walls plays a major role in the stomatal bio-mechanical functionality. The stomatal cellulose microfibrils orientation along the circumference of the guard cell (Ziegenspeck, 1938) forces the elongation of the guard cells that drives pore opening.

Over the years, vast experimental efforts have been invested in characterizing stomatal guard cell structural properties, including geometric parameters (Copeland, 1902; Meckel et al., 2007), material-level composition and micro-structural organization (Palevitz and Hepler, 1976; Zeiger and Hepler, 1976; Stevens, 1977; Mishkind et al., 1981; Jones et al., 2003, 2005; Shtein et al., 2017). In addition, stomatal functionality, i.e., the effects of water pressure on stomatal opening characteristics such as the aperture and the pore opening, have also been extensively studied (Franks et al., 1998, 2001; Franks, 2003; Franks and Farquhar, 2007). In parallel with these experimental investigations, mechanical models that attempted to correlate between the stomatal architecture and its deformation were developed both by analytical means (Aylor et al., 1973; DeMichele and Sharpe, 1973; Sharpe and Wu, 1978; Wu and Sharpe, 1979; Wu et al., 1985) and by numerical simulations (e.g., Finite-Element, FE) (Cooke et al., 1976, 1977; Rui et al., 2016; Carter et al., 2017; Woolfenden et al., 2017). Despite the scientific consensus that guard cells possess high structural-mechanical anisotropy (that is crucial for stomatal functionality), some of these modeling approaches were based on the inappropriate assumption of mechanical isotropy. Analytically, a series of theoretical works (Sharpe and Wu, 1978; Wu and Sharpe, 1979; Wu et al., 1985) aimed to develop a new kinematic framework for the stomatal deformations, which incorporated anisotropic thermo-mechanical models. However, the predictions of these models, although they agree with experimental observations, are based on contradictory mechanical characteristics (e.g., elastic moduli of ~0.1[*MPa*], which are far below the typical values of each of the constituent bio-materials); thus, the model assumptions and its parameters should be refined. In general, an exact rigorous analytic approach to an intricate bio-mechanical system with inherent complexities, such as structural non-uniformity and non-linear deformations, is naturally highly limited compared with numerical simulations.

The results of early numerical attempts to analyze the stomatal behavior (Cooke et al., 1976, 1977) do not coincide well with experimental bio-mechanical observations of stomatal kinetics; for instance, stomatal opening in these studies was driven by cross-sectional deformations, rather than by changes in length. Later studies abandoned the numerical approach for almost four decades, until it was recently revisited in a series of experimentally based FE simulations, which aimed to isolate the key structural, compositional, and mechanical parameters of the guard cell that govern stomatal opening. Recently, Rui et al. (Rui and Anderson, 2016; Rui et al., 2016) used mutants to analyze the effect of variations in cellulose and in the inter-cellulose xylogucan on stomatal opening; these effects were implemented in their FE models via effective changes in the mechanical anisotropy of the guard cell wall. They found that the loss of xyloglucan, which effectively increase the mechanical stiffness anisotropy, facilitates stomatal opening, while cellulose deficiency, which effectively reduces the mechanical anisotropy, has a complementary effect and reduces stomatal opening. In addition, they identified that radial gradients in the wall thickness of the guard cells have a minor effect on the stomatal opening, although it reduces the stress concentration around the pore center. Nevertheless, the simulations performed in these studies introduced the mechanical anisotropy of the cell wall via constitutive relations, which are regulated only for the continuum-level, whereas the direct effects of variations in the bio-composite properties (e.g., cellulose and xyloglucan) on stomatal behavior could not be explicitly identified. Carter et al. (2017) used atomic force microscopy measurements to examine stiffness gradients along the radial direction and epidermal circumference of the guard cells in both young and mature cells, and correlated these gradients with changes in stomatal opening. Their observations indicated that variations in the gradient of radial stiffness do not promote significant differences in stomatal opening, as was also validated by corresponding FE simulations; however, these variations lead to a reduction in stresses at the high-stress regions. Conversely, polar stiffening, which was associated with pectin modifications in these regions, was found to significantly increase stomatal opening, as was also conceptually suggested by Aylor et al. (1973), and this observation was confirmed by effectively pinning (or fixing) the stomatal edges in the FE simulations. Notably, such polar modification at the bio-composite level was also recently found in terms of localized lignification and high crystallinity of the cellulose microfibrils at these regions (Shtein et al., 2017). Woolfenden et al. (2017) used FE simulations to investigate the effct of the mechanical anisotropy of the cell wall, its radial aspect ratio, and the external epidermis pressure on stomatal opening. Their main numerical contribution to previous works lies in explicitly incorporating different materials for the cellulose fibers and matrix, rather than using an effctively constitutive behavior for the entire bio-composite cell wall, which allows examining the specific effct of each phase on the opening characteristics. Their first prime observation is that cell wall anisotropy is esential for stomatal functioning, and that this effect is achived by the circumferential cellolose microfibrils. Another highly important observation was that the non-linear aperture-pressure stomatal behavior originates from strain stiffening of the matrix of the cell wall bio-composite. In addition they identified that the presence of external pressure and the radial architecture (i.e., cell wall thickness and cross-sectional depth-to-width ratio) have a minor effct on the final stomatal opening.

A conclusive qualitative agreement, which emerges from the various studies on stomatal structure-mechanics-function over the years and for numerous stomatal species analyzed via the entire spectrum of scientific methods (analytical, numerical, and experimental), is that the mechanical anisotropy of the guard cells is the main feature that governs the stomatal opening functionality. Nevertheless, to the best of our knowledge, a quantitative investigation of this critical aspect is yet to be conducted, and the relations of such “effective” cell wall mechanical anisotropy to its bio-composite characteristics (i.e., the mechanical properties of the matrix and microfibrils, and their relative content in the cell wall) are yet to be framed. Addressing these pending issues is the main goal of the present study.

This work aims to quantitatively explain how the degree of mechanical anisotropy of the guard cell and its bio-composite properties promote stomatal opening. We thus identify the fundamental, non-dimensional, mechanical parameters that characterize the mechanical anisotropy and use FE simulations to establish general analytical relations between these parameters and the stomatal opening characteristics, i.e., relations that are not restricted to a specific simulation model or to a specific stomatal species. Then, we employ mechanical models for biological materials (Bar-On and Wagner, 2013) to correlate these parameters with the bio-composite, and, thereby, present an analytical explanation of the role of each bio-composite phase (cellulose microfibrils and matrix material) on the stomatal aperture and opening area. These analytical relations quantitatively illuminate the effect of the structure and composition of on stomatal opening, and they complete the extensive recent studies in the field (Jones et al., 2003, 2005; Rui and Anderson, 2016; Rui et al., 2016; Carter et al., 2017; Woolfenden et al., 2017).

Below, section Modeling introduces the two-scale structural and mechanical modeling of the guard cell, i.e., a continuum anisotropic model for the guard cell wall and a composite-materials model for the bio-composite comprising the cell wall, and their implementations in the FE platform. section Stomatal Deformation and Mechanics provides analyses of the guard cell displacement fields, which form the basis for the analytical relations between stomatal opening characteristics, the driving force and anisotropy degree of the guard cell, and its bio-composite mechanical parameters. section Stomatal Bio-mechanics—Additional Functional Insights presents the realization of the developed model by introducing into it the mechanical characteristics typical of plant tissues, and discusses stomatal bio-mechanical functionality in the context of previous experimental works.

## Modeling

### Structural modeling

**Figure 2:**
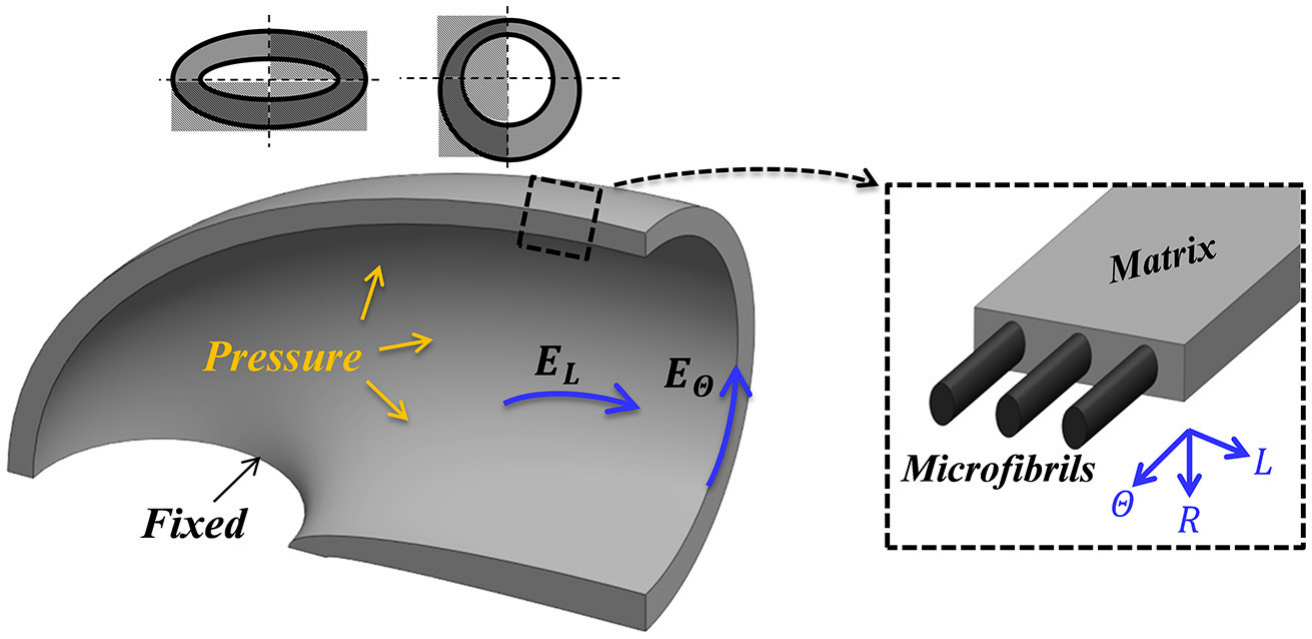
Mechanical guard cell model. One quarter of a guard cell with two symmetry planes, a fixed base and a uniform internal pressure (*P*) is considered for the analysis. The guard cell wall is a composite of circumferentially oriented microfibrils and a matrix that is macroscopically characterized by three elastic moduli (longitudinal, circumferential and radial—*E*_*L*_, *E*_Θ_ and *E*_*R*_, respectively).

Figures 1A,B show micrographs of typical kidney-shaped stomatal structures from *Nephrolepis exaltata* (fern, a relatively primitive plant) and *Tradescantia pallida* (angiosperm, a flowering plant). Structurally, the stomata of the two plants are very similar despite the large evolutionary distance between these plants. At its most basic level, the stoma architecture includes a pair of curved guard cells connected at their ends. Based on the well-accepted model of stomatal geometry (Sharpe and Wu, 1978), each guard cell is a tubule-like element with non-uniform wall thickness, *W*, and, tapered from the center toward the edges, with a varying diameter, *d*. In the leaf-surface plane, the stoma can be viewed as two concentric ellipses: an inner ellipse that encloses the stomatal pore and an outer ellipse that outlines the stomatal margins (Sharpe and Wu, 1978) (Figure 1A). As shown, the in-plane stomatal architecture incorporates the major and minor axes of the inner and outer ellipses [(*x*_0_, *y*_0_) and (*X*_0_, *Y*_0_), respectively], where *X*_0_−*x*_0_ = *d*_*x*_ and *Y*_0_−*y*_0_ = *d*_*y*_ are the tubule diameters along the stomata axes. Guard cell architecture is conveniently characterized by a curved, cylindrical coordinate system whose origin is located at the center of the tubule edge, a longitudinal coordinate (*L*) that follows the center line of the tubule, and circumferential (Θ) and radial (*R*) directions that span the tubule cross-section. In this coordinate system, the localized tubule diameter is merely a function of *L*, and *d*(*L*) ranges between *d*(0) = *d*(π) = *d*_*x*_ and *d*(π/2) = *d*_*y*_. Moreover, to simplify the modeling process, tubule wall thickness is assumed to obey the form *W* = *W*_0_+(*W*_π_−*W*_0_)·Θ/π, which does not vary in the longitudinal direction (Figure 1B). As can be seen, this geometric model includes three symmetry planes (Figure 1C). Subsequent numerical calculations, therefore, can exploit triple mirror symmetry conditions, and as such, they need only consider one quarter of a tubule to enhance the numerical efficiency and computation time of the simulations (Figure 2). The specific geometric parameters used in this work are based on those of (Sharpe and Wu, 1978) as provided in Table S1 (see supporting information). As indicated in recent works, variations from the specific cross-sectional geometry introduced above have a minor effect on the resulting stomatal opening characteristics (Carter et al., 2017; Woolfenden et al., 2017).

### Mechanical modeling

The fundamental mechanical functionality of the stoma is based on an increase in the stomatal water pressure that, reliant on fixed guard cell edges, results in stomatal deformations that lead to pore opening. Such fixed-edge conditions are suggested to be biologically related to localized cell wall stiffening due to confined matrix modifications (i.e., pectin and lignin) and to the increase in the cellulose crystallinity in the stomatal polar regions (Carter et al., 2017; Shtein et al., 2017). From a mechanical perspective, the fixed-edge conditions are shown experimentally and numerically to be critical to achieving a high stomatal opening functionality (Aylor et al., 1973; Carter et al., 2017).

To implement these conditions, the quarter tubule structural model (Figure 2) was subjected to a uniform internal pressure, *P*, associated with fixed-displacement conditions at the guard-cell edge and symmetric conditions at the remaining sections. Typical experimental pressure values for maximal stomatal opening are up to *P* = 5 [*MPa*] (Franks et al., 1998, 2001; Franks, 2003; Franks and Farquhar, 2007). To examine the sensitivity of the analysis to the fixed boundary conditions assumption, a series of axillary simulations were conducted that includes an additional elastic layer, of a different modulus, between the stomata edge and the fixed plane. These variations in boundary conditions, however, led to only slight variations in the results, and the stomatal edges in the remaining simulations were set to be fixed. To summarize, the stiffening of the polar regions of the stoma (Carter et al., 2017; Shtein et al., 2017) and the hypothesis that the stomatal length remains unchanged during inflation (Zeiger et al., 1987) support the fixed boundary conditions approximation. The external walls of the stomata were assumed to be free from forces. Although the external walls are naturally in contact with neighboring cells and are subjected to epidermis pressure, previous observations indicate that epidermal pressure has a minor effect on the magnitude of the final stomatal aperture (at high pressure) and on the force-pressure trend (at moderate-to-high pressures) (Woolfenden et al., 2017).

As mentioned above, the guard cell wall is a bio-composite made of stiff cellulose microfibrils and a softer matrix material (pectin-rich hemicellulose, with some lignin and structural proteins) (Jones et al., 2005). Due to the lack of knowledge of the explicit mechanical properties of pectin (Gibson, 2012) and how the pectin content explicitly changes the effective properties of the hemicellulose, the mechanical properties of the matrix are henceforth considered as a those of hemicellulose. To compensate for the uncertainty of the pectin properties, a broad range of elastic moduli were considered during the theoretical analysis of the bio-composite level (see section Stomatal Bio-mechanics—Additional Functional Insights); this range of parameters is consistent with the results of previous independent numerical optimization processes for stomata (Woolfenden et al., 2017).

Due to the mechanical anisotropy of its cell wall, the guard cell exhibits high stiffness in the local microfibril direction (circumferential direction, Θ), but higher compliance in the perpendicular directions (*L* and *R* directions). Starting from the elementary Voigt and Reuss composite models (direct and inverse rule of mixtures, respectively) (Hull and Clyne, 1996) and using the typical properties of hemicellulose and cellulose published in the literature (e.g., Salmén, 2004; Gibson et al., 2010; Cintrón et al., 2011), the Young's and shear moduli of the orthotropic cell wall are typically related via: (1) *E*_Θ_≫*E*_*L*_, *E*_*R*_, (2) *E*_*L*_≈*E*_*R*_, and (3) *G*_*L*_, *G*_Θ_, *G*_*R*_≈*E*_*L*_. Based on the composite models, typical ranges for these parameters can be estimated as *E*_*L*_ = 0.1−4 [*GPa*] and *E*_Θ_ = 10−40 [*GPa*]; it is further assumed that the Poisson's ratio of the cell wall is ν≈0.3 in all directions. To isolate the net effect of anisotropy and simplify the analytical formulation, the analysis is kept in the framework of linear constitutive relations, i.e., without stiffening effects. Nevertheless, geometrical non-linearity effects, i.e., shape changes upon deformations, which are essentially present due to the extensive stomatal deformations, were considered in the simulations. Due to this simplified assumption, some discrepancies are expected between the trends of the experimental results and of the simulation results; however, the absolute values are expected to be similar (Woolfenden et al., 2017). This aspect is further discussed in section Stomatal Deformation and Mechanics.

### Non-dimensional mechanical parameters

Generally, the applied internal pressure (*P*) generates the stomatal deformations needed for pore opening while the cell wall stiffness—dominated by the two Young's moduli *E*_Θ_ and *E*_*L*_–resists such deformations. The model could thus benefit from the introduction of another parameter, the normalized pressure, $\hat{P}$, a non-dimensional pressure parameter that reflects the effective *driving force* of stomatal deformations:

(1) $$\begin{array}{r} \hat{P}=\frac{P}{E_{L}} \end{array}$$

Note that for a given pressure, greater wall stiffness will produce effectively lower normalized pressure (lower driving force) and thus milder deformation.

Another key parameter with a marked effect on stomatal deformation is the degree of stiffness anisotropy of the cell wall. Two competing mechanisms, the circumferential and longitudinal extensions of the cell wall (the former decreases and the latter increases pore size) dominate pore opening. For a cell wall that is close to isotropic, the two mechanisms exhibit comparable effects and the resultant extent of pore opening is minor. In an anisotropic cell wall, in contrast, the longitudinal extension dominates, and significant pore opening is obtained (Aylor et al., 1973). To that end, numerical simulations of pore opening should include a non-dimensional *anisotropy* parameter—the modulus ratio, Ê:

(2) $$\begin{array}{r} Ê=\frac{E_{\Theta }}{E_{L}} \end{array}$$

In light of the typical ranges of *P*, *E*_Θ_ and *E*_*L*_, the non-dimensional parameters *Ê* = 4−40 and ${\hat{P}}_{\max}\;=0.05$ are considered.

### Finite element implementation

Commercial FE software (ABAQUS 6.12) was used for the numerical simulations. A non-linear solver was employed to account for geometric non-linearity effects due to the large deformations. The quarter tubule guard cell structure was modeled as an elastic orthotropic material (realized via 3-D, 8-node, reduced integration hexahedral elements; C3D8R in ABAQUS element library) with a fine mesh of 70,000 elements. Model boundary conditions, i.e., its fixed and symmetry planes, were set according to Figure 2. Pressure was gradually applied to the inner surface (Figure 2), and the resultant deformations were recorded for each pressure step.

## Stomatal deformation and mechanics

### The effect of driving force and anisotropy on the guard cell deformation patterns

**Figure 3:**
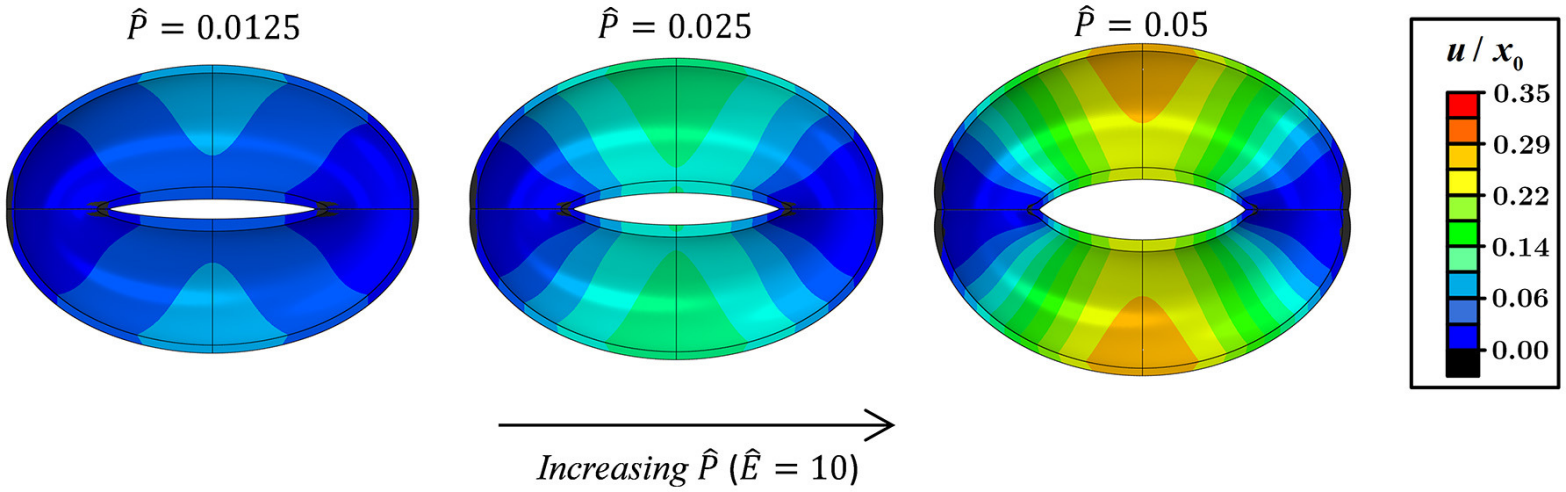
Displacement maps of stomatal opening normalized according to *x*_0_ for increasing $\hat{P}$-values with *Ê* = 10 (see also Supplementary Video S1).

**Figure 4:**
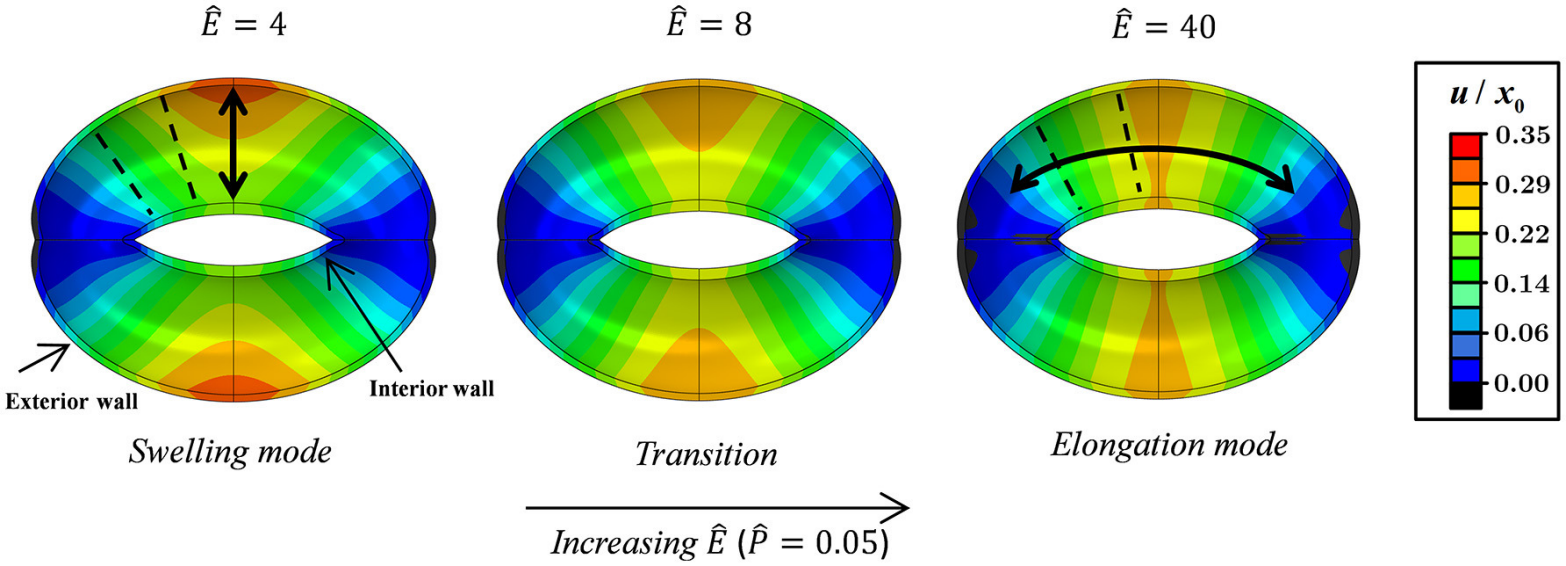
Displacement maps of stomatal opening normalized according to *x*_0_ for increasing Ê-values with $\hat{P}=0.05$. Showing the swelling, transition, and elongation modes (see also Supplementary Video S2). Interior and exterior walls are indicated by arrows. Selected guard cell cross sections are illustrated by dashed lines.

Figures 3, 4 schematically display the guard cell vertical displacement fields, i.e., along the *y* direction (Figure 1), associated with stomatal pore opening. The deformation fields are shown both for varying $\hat{P}$-values (Ê = *const*) to illustrate the effect of the driving force (Figure 3; see also Supplementary Video S1) and for varying Ê-values ($\hat{P}=const$) to illustrate the effect of cell wall anisotropicity (Figure 4). As seen in Figure 3, increasing $\hat{P}$ causes the magnitude of the displacement field to increase monotonically while the field topography remains qualitatively unchanged. In contrast, varying *Ê* results in substantial variations in the displacement field topography (Figure 4; see also Supplementary Video S2). At low Ê-values (e.g., *Ê* = 4), a significant difference is observed between the exterior and interior guard cell walls in each cross section (dashed lines), which means that the guard cell was swelling. At high Ê-values (e.g., *Ê* = 40), however, the cross sections experience approximately uniform displacements, and as such, the vertical movement of each cross section can be viewed as similar to that of a rigid body. The transition between these two extremes is illustrated for *Ê* = 8 (Figure 4). Note that during deformations, the guard cell experience extensive longitudinal strains that may reach even up to 40% at specific regions of the guard cell.

### The dependency between stomatal opening characteristics: aperture and the pore area

**Figure 5:**
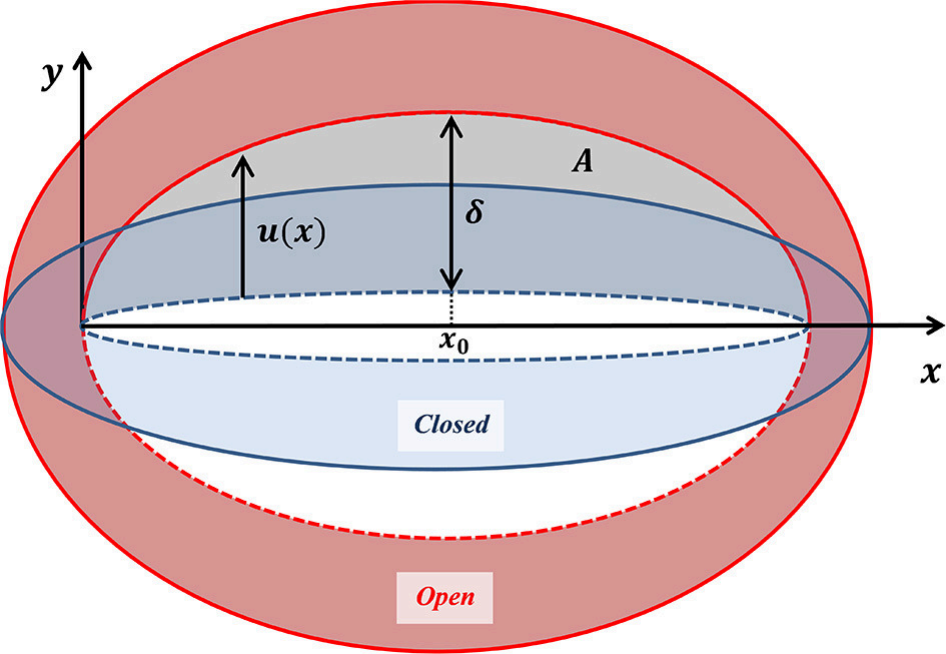
Schematic illustration of a guard cell in its closed (blue) and open (red) states, shown with the corresponding displacement of the interior wall of the guard cell (as indicated in Figure 4). The localized displacement *u*(*x*) due to pore opening, and the aperture δ≡*u*(*x*_0_), are indicated by the black arrows. The area of a pore $A\equiv \int_{0}^{2x_{0}}u\left(x\right)dx$ is indicated in gray.

While the guard cell displacement field represents overall stomatal deformation, the vertical displacement of the interior wall of the guard cell (indicated in Figure 4)—the displacement *u*(*x*) associated with pore opening—in fact determines the stomatal functionality (Figure 5). Two experimental parameters are commonly employed to evaluate the effectiveness of stomatal opening: (i) the stomatal pore aperture δ≡*u*(*x* = *x*_0_)–the displacement of the interior wall in the stomatal middle, and (ii) the net pore opening area $A\equiv \int_{0}^{2x_{0}}u\left(x\right)dx$. On a first sight, these two parameters may appear to be independent. In light of the guard cell symmetry (around *x*_0_) and its fixed edge conditions (at *x* = 0, 2*x*_0_), *u*(*x*) can be generally expressed via:

(3) $$\begin{array}{r} u\left(x\right)=\delta \cdot \left[\frac{x}{x_{0}}\cdot \left(2-\frac{x}{x_{0}}\right)\cdot f\left(x\right)\right] \end{array}$$

where *f*(*x*) is an even function that satisfies *f*(*x* = *x*_0_) = 1. A noteworthy outcome of this theoretical representation is that δ and *A* are in fact tightly related in a linear fashion—disregarding the magnitude of the driving force ($\hat{P}$) and the specific shape of stomatal deformations (*u*(*x*)):

(4) $$\begin{array}{r} A/\delta =k\left(x_{0}\right) \end{array}$$

This formula establishes a linear relationship between the stomatal pore opening area and the pore aperture. Interestingly, this relationship is based mainly on simple geometry.

**Figure 6:**
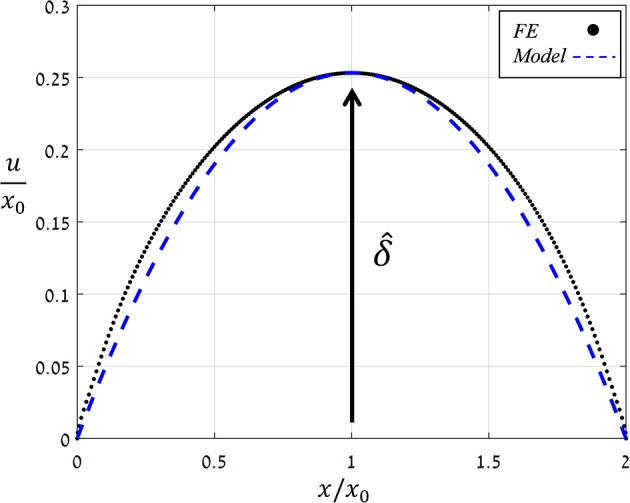
Normalized opening displacement along the stoma (*u*/*x*_0_) vs. the normalized location (*x*/*x*_0_) as obtained by FE simulations for $\hat{P}=0.05$ and *Ê* = 10 (black dots) and by simplified theoretical modeling of a parabolic opening displacement (blue dashed line). The normalized aperture $\hat{\delta }=\delta /x_{0}$ is indicated. The same trend was obtained for all Ê- and $\hat{P}$-values analyzed.

**Figure 7:**
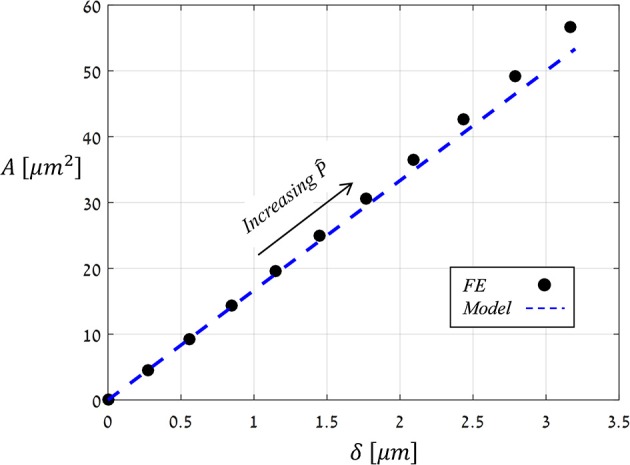
FE results for the stomatal aperture, δ, vs. the opening area, *A* (black dots), as obtained by increasing $\hat{P}$ (for *Ê* = 10), and theoretical modeling results of a simplified parabolic opening displacement (blue dashed-line). Note the linear relation between *A* and δ, which was obtained for all Ê-values analyzed.

Figure 6 shows the displacement due to pore opening *u*(*x*) obtained from FE simulations for $\hat{P}=0.05$ and *Ê* = 10. Note the strong compatibility between the theoretical model of a simplified parabolic form, Equation (3) with *f*(*x*) = 1, and the simulation results. The same degree of compatibility emerges for the entire range of $\hat{P}$ and *Ê* tested. An additional series of simulations were conducted in which the values of $\hat{P}$ were increasing while those of *Ê* were fixed, and from each simulation, values for δ and *A* were extracted (Figure 7) and shown to be linearly related. For comparison, the resultant theoretical ratio of the parabolic model, *A*/δ = 4/3 · *x*_0_, is also plotted in Figure 7. This simplified approximation clearly agrees with the FE simulations. Note that the above theoretical prediction for the *A*/δ ratio is in agreement (up to 20% deviation) with that of previous experimental work (Meckel et al., 2007).

### The effects of driving force and anisotropy ratio on the stomatal aperture

**Figure 8:**
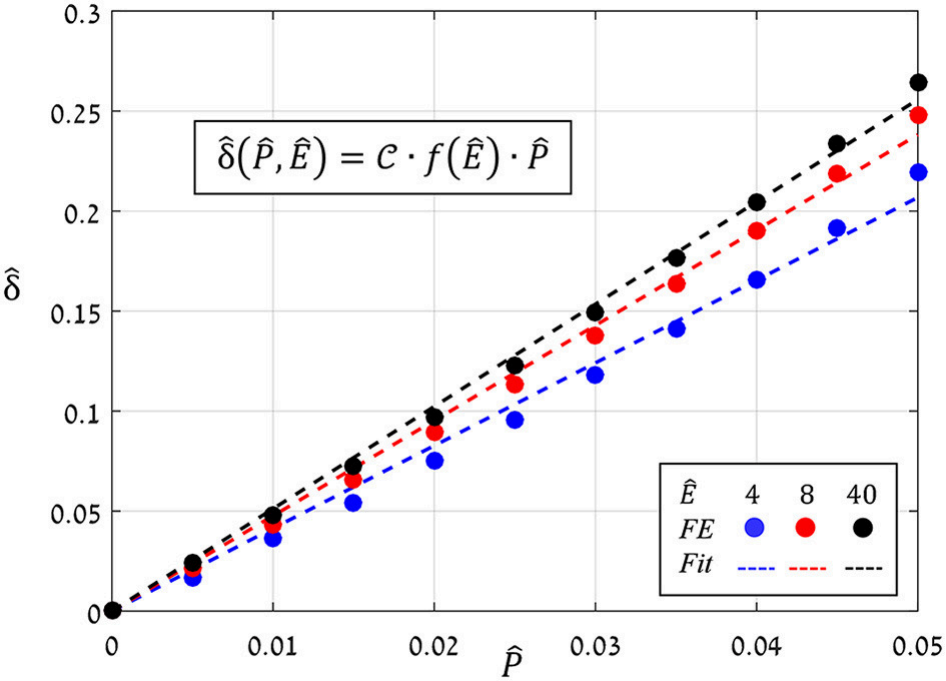
FE results of $\hat{\delta }$ vs. $\hat{P}$ for *Ê* = 4, 8, *and* 40 (blue, red and black circles, respectively); the corresponding linear fittings are indicated by the dashed lines.

The preceding analysis focused on the normalized aperture, $\hat{\delta }=\delta /x_{0}$, which conveniently indicates the magnitude of pore opening (Figure 6). To determine the analytical dependence of $\hat{\delta }$ on $\hat{P}$ and Ê, a series of simulations were run in which values of $\hat{P}$ were increasing ($0\to {\hat{P}}_{\max}=0.05$) while values of *Ê* were fixed, and from each simulation, the resultant aperture $\hat{\delta }\left(\hat{P};Ê\right)$ was recorded. The simulations results for *Ê* = 4, 8, and 40 (Figure 8) show that:

(5) $$\begin{array}{r} \hat{\delta }\left(\hat{P},Ê\right)=C\left(Ê\right)\cdot \hat{P} \end{array}$$

**Figure 9:**
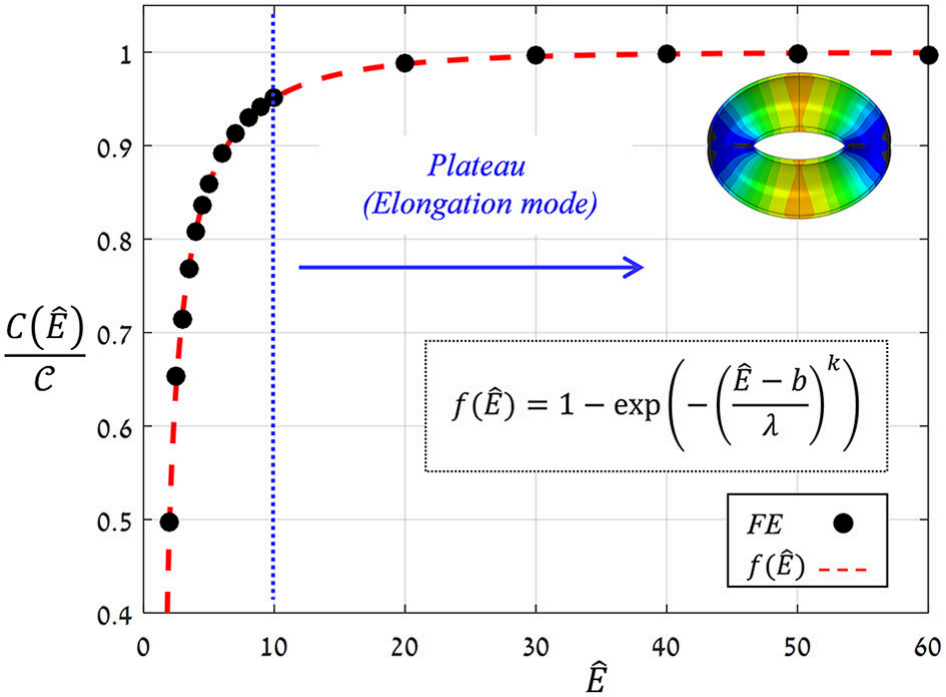
FE results for *f*(Ê) (black circles) and their exponential fitting (red dashed-line); fitting parameters are *k* = 0.48, *b* = 1.6, λ = 0.8. A plateau of *f*(Ê)≈1 that correlated with the guard cell elongation mode was obtained for Ê≥10 (blue lines). The threshold Ê^*^ = 10, above which guard cell deformation was in elongation mode, is indicated by the blue line; a representative displacement map of stomatal opening in elongation mode (for *Ê* = 40>Ê^*^) is displayed.

The proportion ratio *C*(Ê) can be interpreted as the macro-structural compliance of the guard cell. Interestingly, despite the considerable shape change undergone by the guard cell upon inflating, a clear linear $\hat{\delta }-\hat{P}$ trend was obtained for the entire range of *Ê* tested. Thus, the stomatal aperture is linearly related to internal turgor pressure. Next, to identify the functional form of *C*(Ê), the corresponding $\hat{\delta }-\hat{P}$ slopes were extracted from each set by linear fitting. These slopes were found to approach a maximal value C, i.e., $C(\hat{E}\gg 1)\to C$, which represents the maximal macro-mechanical compliance of the guard cell (Figure 9). Guard cell compliance can thus be conveniently formulated as:

(6) $$C(\hat{E})=C\cdot f(\hat{E})$$

where the functional form of *f*(Ê) represents the net effect of guard cell anisotropy. Figure 9 shows the values of C(Ê)/C from the FE simulations, from which the analytical form of *f*(Ê) was extracted (using linear fitting in logarithmic scales Supplementary Figure S1). To identify the functional form of *f*(Ê), the range of *Ê* analyzed was temporarily extended beyond the tested range of *Ê* = 4−40 (using *Ê* = 2, 2.5, 3, 3.5, and *Ê* = 50, 60). The resultant *f*(Ê) was found to be well-described by the following three-parameter Weibull-like exponential form with scale, shape and shifting parameters (λ, *k* and *b*, respectively):

(7) $$\begin{array}{l} C\left(Ê\right)/C\;=f\left(Ê\right)=1-\exp\left(-{\left(\frac{Ê-b}{\lambda }\right)}^{k}\right) \end{array}$$

This functional form of *f*(Ê) shows that a certain threshold value for Ê^*^ can be readily identified, and beyond that value, a plateau is reached and maximum guard cell compliance is achieved $C(\hat{E}>{\hat{E}}^{*})\approx C$ [Equation (6)]. This threshold can be estimated by letting *f*(Ê^*^)≥ 1−exp(−*n*), where the magnitude of *n* dictates the specific threshold value via ${\hat{E}}^{*}\geq \lambda \cdot \sqrt[{k}]{n}+b$. It can be seen that for *n* = 3, Ê^*^ ≈10, beyond which *f*(Ê)>0.95 (Figure 9); note that for *n* = 4, Ê^*^ ≈15 and *f*(Ê>Ê^*^)>0.98. It is worth noting that the threshold for plateau initiation (Ê^*^) also marks the beginning of the elongation-dominant mode of guard cell deformation (shown in Figure 4).

It is worth mentioning that this “plateau” holds various cases of stomatal wall compositions (e.g., the presence of lignin and pectin) and the crystallinity degree of the components (e.g., the crystallinity of the cellulose microfibrils may differ along the guard cell). While these effects may significantly change the Young's modulus of both the matrix and the microfibrils, the resultant modulus ratio will still be higher than Ê^*^. Thus, the stomatal architecture is apparently a highly robust element which is insensitive to such variabilities in the stomatal cell wall composition—and its mechanical behavior is effectively characterized by the asymptotic compliance C.

In summary, the stomatal opening area and its normalized aperture are analytically dependent on the driving force and anisotropy parameters ($\hat{P}$ and Ê) via:

(8) $$\begin{array}{l} A\left(\hat{P},Ê\right)\propto \hat{\delta }\left(\hat{P},Ê\right)=C\cdot \left\{1-\exp\left[-{\left(\frac{Ê-b}{\lambda }\right)}^{k}\right]\right\}\cdot \hat{P} \end{array}$$

where C=5.1 and where *k* = 0.48, *b* = 1.6, *and λ* = 0.8 and are assumed to be dependent merely on guard cell geometry. The parameter *b* is the minimum value of *Ê* for which the correlation is adequate, and this effective value represents the minimum anisotropy of the stomatal wall that is essential to generate stomatal opening. For Ê>Ê^*^, Equation (5) reduces to the following form, for which maximal stomatal opening (area and aperture) is obtained:

(9) $$\begin{array}{l} A\left(\hat{P}\right)\propto \hat{\delta }\left(\hat{P}\right)\approx C\cdot \hat{P} \end{array}$$

As mentioned above, in the present analysis we considered non-linear effects in the form of shape changes, and kept the material constitutive law to be elastic linear. To adequately extend the FE simulations to consider material non-linearity effects, an experimentally based constitutive law of the matrix material must be explicitly introduced. To the best of our knowledge, such constitutive relations cannot be found for the pectin-rich hemicellulose matrix material. To explain the matrix stiffening effects, recent studies (Carter et al., 2017; Woolfenden et al., 2017) incorporated fundamental parametric models, which were originally developed for rubbery engineering materials (Rivlin and Saunders, 1951) or for skin (Veronda and Westmann, 1970), into the FE simulations, and employed an optimization process to minimize the discrepancies with experimental results (Woolfenden et al., 2017). To account for these non-linear effects from an analytical perspective, the formula in Equation (5) can be straightforwardly generalized by including a series expansion, e.g., polynomial $A\propto \hat{\delta }\approx C_{1}\left(Ê\right)\cdot \hat{P}+C_{2}\left(Ê\right)\cdot {\hat{P}}^{2}+\ldots C_{n}\left(Ê\right)\cdot {\hat{P}}^{n}$, and to employ parameter fitting to experimental results. Clearly, as such a series expansion completely spans the analytical function space, one could always find an expansion that adequately follows any experimental result. Nevertheless, since there are only limited experimental results with explicit force-pressure relations, and since the deviation of the results with and without matrix stiffening effects are relatively small (Woolfenden et al., 2017), such a generalization of the analytical model will provide substantial and conclusive insights beyond those that were already introduced above.

### Relations to the bio-composite mechanics

From a material-level perspective, the guard cell wall is viewed as a bio-composite of circumferential microfibrils (cellulose), with Young's modulus *E*_*f*_ and volume fraction ϕ, which are surrounded by a pectin-rich hemicellulose matrix material with Young's modulus *E*_*m*_. By using the fundamental Voigt and Reuss composite models (Hull and Clyne, 1996), the effective modulus of the guard cell in the circumferential and longitudinal directions can be estimated:

(10a) $$\begin{array}{l} E_{\Theta }=\phi \cdot E_{f}+\left(1-\phi \right)\cdot E_{m} \end{array}$$

(10b) $$\begin{array}{l} E_{L}={\left[\phi /E_{f}\;+\left(1-\phi \right)/E_{m}\;\right]}^{-1} \end{array}$$

By incorporating these relations into Equations (1)—(2) and by using generally accepted bio-composite characteristics [i.e., *E*_*f*_ ≫ *E*_*m*_, ϕ ~ *O*(0.1)], $\hat{P}$ and *Ê* can be approximated as:

(11a) $$\begin{array}{l} \hat{P}=P/E_{L}\;\approx P\cdot \left(1-\phi \right)/E_{m} \end{array}$$

(11b) $$\begin{array}{l} Ê=E_{\Theta }/E_{L}\;\approx E_{f}\;\cdot \phi \cdot \left(1-\phi \right)/E_{m} \end{array}$$

A number of important insights can be drawn from these relations. Firstly, although matrix stiffness has a marked effect on the driving force $\hat{P}$, the effect of microfibril stiffness on $\hat{P}$ is negligible. This finding hints at the possible functional importance of differences in the stomatal cell wall compositions of various plant species (Jones et al., 2003, 2005; Shtein et al., 2017).

Secondly, the value of the anisotropy parameter is set according to the ratio *E*_*f*_/*E*_*m*_ that, in turn, dictates which deformation mode will be observed, e.g., *E*_*f*_/*E*_*m*_ < 40 produces swelling modes and *E*_*f*_/*E*_*m*_>400 generates elongation modes. The transition region between these two extremes is obtained in the vicinity of Ê^*^ ~10, i.e., for *E*_*f*_/*E*_*m*_ ~*O*(100); this order of magnitude between the cellulose-to-matrix modulus ratio has also been identified in an independent numerical optimization process (Woolfenden et al., 2017). This finding shows that the model is in good agreement with the empirical findings, i.e., cellulose microfibrils are the load-bearing (stiff) portion of the cell wall, and the matrix compliance is essential to the ability of stomata to open (an overly too “stiff” matrix will produce a minor opening). Interestingly, the fibers-to-matrix stiffness ratio is not important as long as it is >Ê^*^ ~10, meaning that above the threshold value for *E*_*f*_ there is no “gain” for stiffer fibers—namely, the maximal compliance is achieved and a maximal aperture for a given pressure is reached. These bio-composite level interpretations, i.e., the key role of the matrix compliance and of a threshold value for the fibers stiffness inferred from the above analytical formulations, also emerged in recent experiment-based numerical works (Carter et al., 2017; Woolfenden et al., 2017).

## Stomatal bio-mechanics—additional functional insights

**Figure 10:**
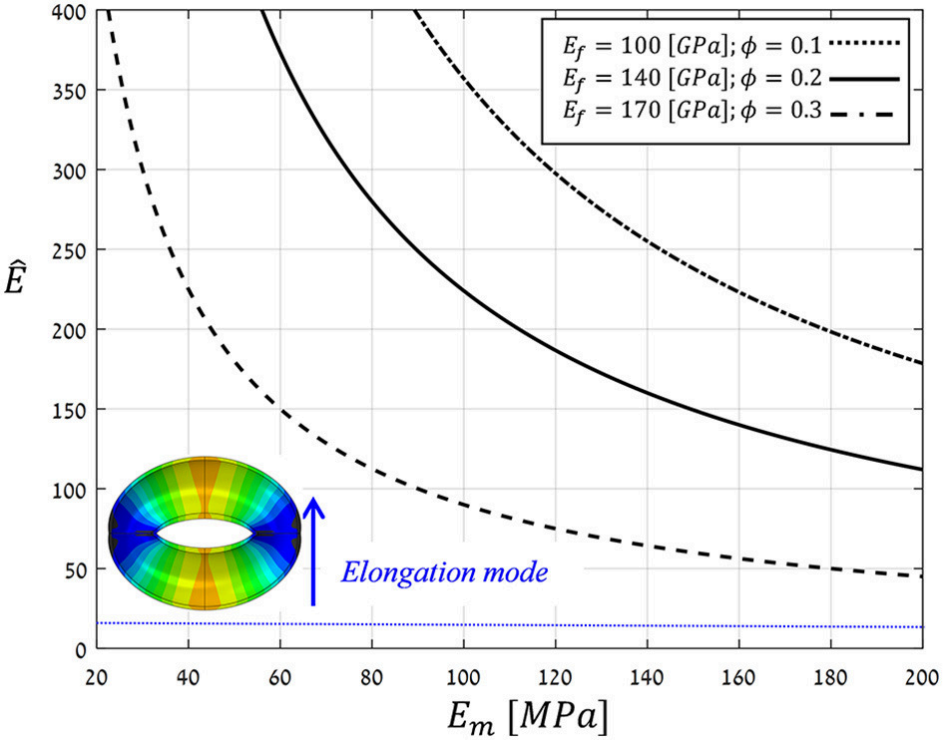
Theoretical estimations for the anisotropy parameter *Ê* calculated by Voigt and Ruess using composite models for a range of typical Young's moduli of cellulose microfibril, hemicellulose matrix, and microfibril contents in living plant tissues, i.e., wet conditions (*E*_*f*_, *E*_*m*_ and ϕ, respectively) (Salmén, 2004; Gibson et al., 2010; Cintrón et al., 2011; Dri et al., 2013). The threshold Ê^*^ = 10, above which guard cell deformation is in elongation mode, is indicated by the blue line; inset shows a representative opening displacement field in elongation mode (for *Ê* = 40>Ê^*^).

To realize the above multi-scale, structural-mechanical modeling for guard cell deformations, typical bio-composite characteristics of living plant tissues (i.e., wet conditions) were employed for material modeling: *E*_*f*_ = 100−170 [*GPa*], *E*_*m*_ = 20−200 [*MPa*], and ϕ = 0.1−0.3 (Salmén, 2004; Gibson et al., 2010; Cintrón et al., 2011; Dri et al., 2013). Figure 10 shows the resultant anisotropy parameter (Ê) as calculated for the above parameter ranges. Note that for the entire parametric range analyzed, $Ê\left(E_{f},E_{m},\phi \right)\gg {Ê}^{*}\;\approx 10$, indicating that for realistic bio-mechanical characteristics:

(a) the guard cell deformation mode is essentially elongation. Interestingly, this analytical outcome coincides with a well-established hypothesis in stomatal mechanics, namely, that the circumferential alignment of the microfibrils confers significant mechanical anisotropy on the cell that, in turn, enhances stomatal opening capabilities (Aylor et al., 1973).

(b) the stomatal aperture and opening area can be analytically represented by Equation (9). The incorporation of Equation (11a) into Equation (9) facilitates the explicit expression of the guard cell aperture and the opening area by the internal pressure (*P*), the fiber content (ϕ) and Young's modulus of the matrix (*E*_*m*_):

(12) $$\begin{array}{l} A\left(E_{m},\phi \right)\;\propto \;\delta \left(E_{m},\phi \right)\;\propto \;P/E_{m}\cdot \left(1\;-\;\phi \right) \end{array}$$

**Figure 11:**
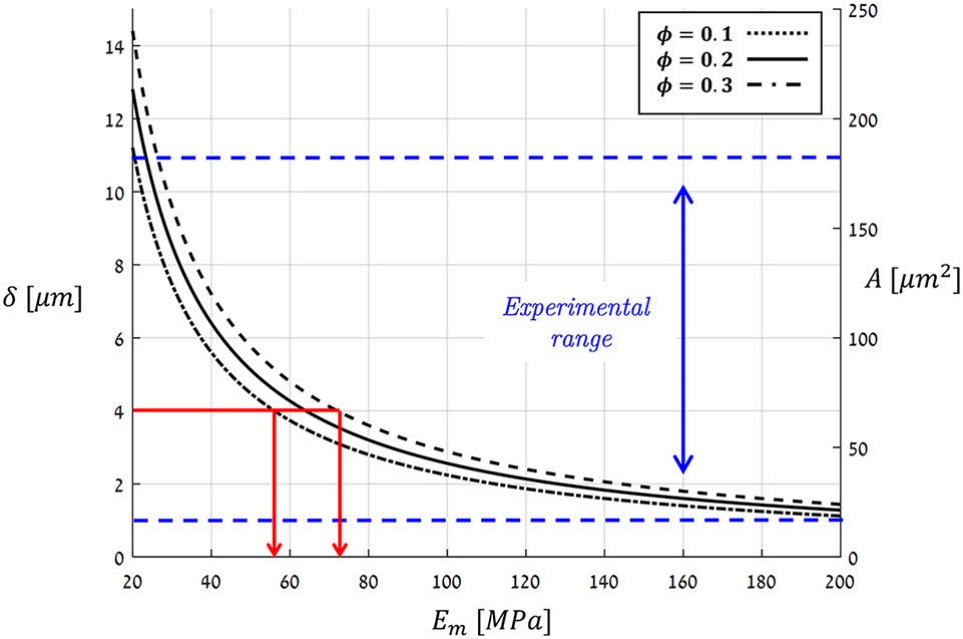
Theoretical predictions for the stomatal aperture (δ) and pore area (*A*), as calculated by Equation (12) at a maximal pressure state (*P*_max_ = 5 [*MPa*]) for a typical range of *E*_*m*_- and ϕ-values (living plant tissues, i.e., wet conditions). The range for experimentally measured *A*- and δ-values for various species of guard cells from literature is indicated by the blue dashed-lines (summarized in Table 1). Red lines—the estimation of *E*_*m*_ for *Ginkgo biloba*.

Figure 11 plots the theoretical predictions of Equation(12) for *P* = *P*_max_ = 5 [*MPa*] using the typical bio-composite characteristics cited above for *E*_*m*_ and ϕ, which resulted in an aperture range of δ = 1 → 15 [μ*m*] and an opening area range of *A* = 20 → 250 [μ*m*^2^]. These predictions are based on a vast collection of experimental data representing stomata from a wide variety of plant species (Figure 11, Table 1). As can also be seen in Figure 11, *A* and δ rapidly decrease with increasing *E*_*m*_. For hypothetical *E*_*m*_-values that correspond to hemicellulose under dry plant tissue conditions (i.e., *E*_*m*_≈2, 000−8, 000 [*MPa*]), the theoretical predictions yield δ < 0.15 [μ*m*] and *A* < 2 [μ*m*^2^] (not shown in Figure 11), which are far below the experimental range. Because photosynthesis does not occur in dry, dead leaves, such small range values for stomatal aperture and opening area are to be expected. Thus, we are able to predict stomatal opening parameters using material structural properties (based on the mechanical characteristics of the guard cell wall constituents).

**Table 1:** Experimental datafrom literature for the opening area and aperture of stomata of various plant species, adapted for the geometricaldefinitions in Figure 5.

**Species**	**Source**	**Area [*μm*^2^]**	**Aperture [*μm*]**
*Aspidium acrostichoides*	[1]	–	≈ 3.5
*Azalea nudiflora*	[1]	–	≈ 1.5
*Botrychium ternatum*	[1]	–	≈ 1
*Dennstaedtia punctilobula*	[1]	–	≈ 1.5
*Funaria hygrometrica*	[1]	–	≈ 1
*Ginkgo biloba*	[2]	–	≈ 4
*Huperzia prolifera*	[3]	≈ 25	≈ 2−2.5
*Lycopodium lucidulum*	[1]	–	≈ 1
*Medeola virginica*	[1]	–	≈ 2
*Nephrolepis exaltata*	[2]	–	≈ 3.5
	[3]	≈ 75	≈ 2
*Tradescantia virginiana*	[2]	–	≈ 4.5
	[3]	≈ 350	≈ 2.5−10
*Vicia faba*	[2]	–	≈ 4
	[4]	–	7.5
	[5]	≈ 70	≈ 3
	[6]	–	≈ 4−11

[1] , Copeland (1902);

[2] , Franks et al. (1998);

[3] , Franks and Farquhar (2007);

[4] , Sharpe and Wu (1978);

[5] , Meckel et al. (2007);

[6] *, Franks et al. (2001)*.

In a complementary viewpoint, the theoretical results in Figure 11 span an interesting applicative aspect for bio-material science—to inversely estimate the *E*_*m*_ of the guard-cell bio-composite merely from the stomatal aperture. As an example, for Ginkgo biloba (Franks et al., 1998) with δ = 4 [μ*m*], one can back-calculate the matrix modulus of the guard cell to be *E*_*m*_≈60−70 [*MPa*] (Figure 11, red lines). Such an analysis is highly beneficial by being non-destructive that requires simply microscopic means—and can be conducted directly in *in situ* conditions. To the best of our knowledge, there is yet no other experimental method that can measure the Young's modulus of the matrix material in plants bio-composites, and in particular in guard-cells, in their native state.

## Summary

This work proposes a new analytical-numerical framework for investigating the bio-mechanical functionality of leaf stomata. Two non-dimensional continuum-level parameters that dominate stomatal opening characteristics—the driving force and the anisotropy ratio—were identified, and their analytical relations to stomatal aperture were established. Next, material-level bio-composite models were used to correlate these continuum-level parameters with the mechanical properties of the bio-composite constituents comprising the cell wall; consequently, closed-form analytical formulae that correlate the fundamental mechanical constituents of the guard cell wall with the characteristics of stomatal opening were established. Thus, for cases in which the bio-composite properties of guard cells are at hand, the stomatal aperture and opening area can be directly provided by a simple substitution, without the need of complex numerical simulations. According to the presented analytical model, the stomatal aperture is linearly related to internal turgor pressure $\hat{P}$, and this relation can be straightforwardly generalized, if desired, to adequately describe any experimentally observed stiffening effects. The stomatal compliance is dependent on the non-dimensional mechanical anisotropy Ê; when a threshold Ê^*^ is crossed—the compliance reaches maximal asymptotic value. The cellulose microfibrils constitute the stiff, load-bearing portion of the stomatal cell wall, while soft matrix is essential to the ability of stomata to open. Thus, a stiffer matrix will generate a small opening and will promote swelling deformation mode. These findings may indicate a biomechanical role for differences in stomatal cell wall composition across different plant species.

The predictions of the proposed model were found to be in agreement with the experimental results cited in the literature. In addition, the functional insights gleaned from the elucidated relations between stomatal architecture and mechanics, both at the material level and at the structural level, may pave the way for the development of new types of miniature, bio-inspired valves for micro-mechanical devices and biomedical applications.

## Author contributions

All authors listed have made a substantial, direct and intellectual contribution to the work, and approved it for publication.

### Conflict of interest statement

The authors declare that the research was conducted in the absence of any commercial or financial relationships that could be construed as a potential conflict of interest.
