# Supplementary material for: Stomatal Opening: The Role of Cell-Wall Mechanical Anisotropy and Its Analytical Relations to the Bio-composite Characteristics
Source: Front Plant Sci. 2017 Dec 12;8:2061. doi: 10.3389/fpls.2017.02061 (PMC5733087; doi:10.3389/fpls.2017.02061)
Supplement: Supplementary file 3 [file DataSheet1.DOCX]

**Supporting information**

# Stomatal micro-to-macro mechanics

Ziv marom, Ilana Shtein, Benny Bar-On

| Geometrical  parameters | [25] | [10,19] |
| --- | --- | --- |
| $x_{0}$ | $\approx12.5 [\mu m]$ | $-$ |
| $d_{x}$ | $x_{0}$ | $-$ |
| $d_{y}$ | $\frac{2}{3}\left( x_{0}+d_{x} \right)-y_{0}$ | $-$ |
| $y_{0}$ | $\frac{x_{0}+d_{x}}{50}$ | $-$ |
| $W_{0}$ | $-$ | $\approx1.5 \left[ \mu m \right]$ |
| $W_{\pi}$ | $-$ | $\approx1 \left[ \mu m \right]$ |

Table S1. Model geometrical parameters, used in the present work (see Figure 1), and their corresponding literature origins.


$$X$$

$$Y$$

| *FE* |  |
| --- | --- |
| *Fit* |  |

$$Y=k\cdot(X-\ln\lambda)$$

$$k$$

Figure S1: FE results for $C(\hat{E}\mathcal{)/C}$ (black circles) in Figure 9 after axis scaling $Y=\ln\left[ -\ln\left[ 1-C(\hat{E}\mathcal{)/C} \right] \right]$ and $X=\ln\left[ \hat{E}-b \right]$, exhibited with the corresponding linear fitting (red dashed-line). In this representation, the slope is $k$ and $\lambda=exp(X-Y/k)$.

Video S1: Stomatal deformations for increasing $\hat{P}$, for $\hat{E}=10$.

Video S2: Stomatal deformations for increasing $\hat{E}$, for $\hat{P}=0.05$.
